# Suicides Mortality of Unemployed Individuals Becomes a Serious Public Health Concern in Japan in Post-COVID-19 Pandemic Era

**DOI:** 10.3390/ijerph22091315

**Published:** 2025-08-22

**Authors:** Tomoka Oka, Ryusuke Matsumoto, Eishi Motomura, Motohiro Okada

**Affiliations:** Department of Neuropsychiatry, Division of Neuroscience, Graduate School of Medicine, Mie University, Tsu 514-8507, Japan; tomoka-oka@clin.medic.mie-u.ac.jp (T.O.); matsumoto-r@clin.medic.mie-u.ac.jp (R.M.); motomura@clin.medic.mie-u.ac.jp (E.M.)

**Keywords:** employment, gender, Japan, post-COVID-19 era, suicide mortality, unemployment

## Abstract

Identification of temporal relations among suicide mortality and economic/political implementations provides important information for not only planning suicide prevention but also socioeconomic/psychosocial measures. This cross-sectional observation study analyzed temporal fluctuations and causalities of suicide mortalities of working-age individuals, disaggregated by age/gender/social standing (employed/unemployed), in Japan from 2009 to 2024, using government databases, by joinpoint and vector-autoregressive analyses. Suicide mortality among total and employed females decreased until the COVID-19 pandemic outbreak but sharply increased, synchronized with the pandemic outbreak, before resuming a downward trend. Among males, the decreasing trends attenuated from 2016, followed by a transient increase in 2022. Unemployed males aged 40–69 exhibited four joinpoints: 2016 (decreasing–increasing), 2018 (increasing–decreasing), 2022 (decreasing–increasing), and 2023 (increasing–stable). In contrast, suicide mortality among unemployed females aged 40–69 sharply increased in 2022 and maintained the high level. Among individuals aged 30–39, suicide mortality reversed from decreasing to increasing in 2016 (males) and 2018 (unemployed females). Economic expansion was protective for employed individuals but had no significant effect on unemployed populations. The government management instability (AENROP) index was positively associated with suicide mortality among employed and unemployed males and employed females. Unemployed females aged 30–39 were sensitive to AENROP but not economic conditions, while those aged 40–69 were largely unaffected by either. Increasing employment of individuals with psychiatric disabilities was positively associated with suicide mortality among unemployed males (30–69) and females under 40. Positive impacts of the employment rates of individuals with psychiatric disabilities and unemployment enhanced from 2016 and 2022, respectively, whereas the impacts were inconstantly affected by political rather than economic factors. Suicide mortality among unemployed individuals has emerged as a critical public health concern in Japan, with rates more than doubling among males and tripling among females in the 2020s. These findings underscore the need for integrated suicide prevention policies that address both labor market vulnerabilities and psychosocial determinants.

## 1. Introduction

**Figure 1:**
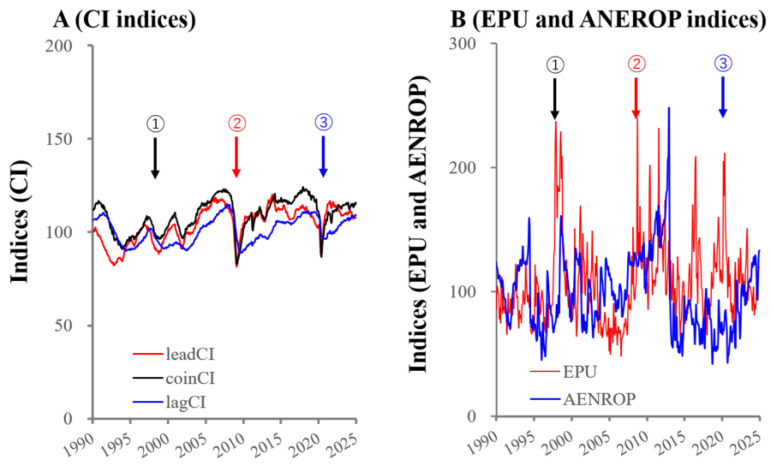
Fluctuations of indices of economic activity and uncertainty in Japan from 1990 to 2024. Left (**A**) and right (**B**) side panels indicate fluctuation of indices of economic activity (CI indices) and political uncertainties (economic policy uncertainty: EPU and government management instability: AENROP), respectively, from January/1990 to December/2024 in Japan. Ordinate and abscissa indicate the indices and calendar years, respectively. Arrows indicate the 1997 Asian financial crisis (No. 1), the 2008 global economic crisis (No. 2), and the COVID-19 pandemic outbreak (No. 3).

The relevant literature supports that economic downturns/recessions contribute to increasing suicides, with greater impacts on males than females due to developed unemployment/economic hardship [1,2,3,4,5,6,7]. The facts historically illustrate this phenomenon, such as the Great Recession from the 1920s to 1930s, the 1997 Asian financial crisis, and the 2008 global financial crisis [8,9,10]. Following the 1997 Asian financial crisis, suicides in Japan also sharply increased (males > females) by >30% in 1998, remaining elevated until 2009 [9,11,12,13,14,15,16]. After the 2008 crisis, which was the largest recession in this century [17,18], numerous studies reported increasing suicides in Western countries [19,20,21,22]. Japan also experienced severe economic downturns during the 2008 crisis [23]; rather, the Economic and Social Research Institute in Cabinet Office (ESRI) reported that the impact of the 2008 crisis was larger than the 1997 Asian financial crisis [24] (Figure 1). However, suicide patterns between the 1997 Asian financial crisis and the 2008 crisis significantly differed, since overall suicides in Japan did not display significant increases after the 2008 global financial crisis [10,23,25]. Unexpectedly, rather suicide mortality in Japan, which had consistently maintained high levels over the decade since the 1997 Asian financial crisis, began decreasing from 2009 [11,26,27]. The COVID-19 pandemic has caused a severe economic downturn comparable to the 2008 crisis, worldwide and also in Japan [24,28] (Figure 1). Various reports in the initial stage of the pandemic were deeply concerned by increasing suicides caused by the COVID-19 pandemic, but major countries in the Organization for Economic Co-operation and Development (OECD) did not increase suicides during the pandemic [29,30,31,32,33,34]. On the contrary, in Japan, the COVID-19 pandemic outbreak only triggered increasing working-age female suicides, without affecting male suicides [35,36,37,38,39,40]. However, the precise mechanisms underlying the exceptionally and specifically increased female suicides during the pandemic in Japan, compared to other OECD member countries, remain to be elucidated.

Suicide has been considered to represent a temporally complex phenomenon with multifaceted risk factors, including individual psychosocial and socioeconomic factors [5,7,41,42,43]. Socioeconomic factors at both the individual and social levels have been intrinsically linked and mutually reinforcing in East Asia, including in Japan [14,44,45,46]. Socioeconomic factors, including unemployment, have been traditionally associated with suicide among East Asian males (including those from China, Taiwan, Japan, Korea, and Hong Kong) and have been interpreted in the context of traditional values due to collective rather than individual values, including the importance of maintaining family [14,44,45,46]; however, sharply increasing female suicides in Japan synchronized with the pandemic outbreak cannot be explained by conventional hypotheses. Many attribute this discrepancy between the genders—which contrasts with historical recession patterns—to complex psychosocial and socioeconomic interactions (including both the direct effects of COVID-19 and government containment measures [36,38]).

Unemployment is an established suicidal risk among economic factors [3,47,48,49], whereas current research suggests that in several high-income/welfare countries, the positive relationship between unemployment and suicide has attenuated to non-significance, largely due to favorable effects of comprehensive unemployment insurance programs [2,19,50,51]. Instead, suicides appear more sensitive to economic policy uncertainty propagated through mass media than to unemployment [50,51,52,53,54,55,56,57]. Furthermore, government-led suicide prevention initiatives have demonstrated significant effectiveness in reducing suicides [7,11,26,27,43,58,59]. Consequently, elucidating causes underlying sharply increasing female suicides, synchronized with the pandemic outbreak, and the subsequent improvement in suicide mortality in the post-pandemic period in Japan could provide important information for planning/refining government suicide prevention strategies in the post-COVID-19 pandemic era in line with evidence-based policymaking. Rather than simply comparing the suicide mortality trends before, during, and after the pandemic in 2009–2024, comprehensive analyses of temporal fluctuations of suicide mortality disaggregated by age, gender, and social standing during 2009–2024 should be conducted to screen high-risk suicide groups [60]. Subsequently, conducting causality analyses to clarify major economic/political factors underlying the increasing suicides in high-risk groups may identify specific risk factors to target in suicide prevention programs. We therefore conducted time series and causality analyses using government databases to clarify temporal fluctuations and the causality of crude suicide mortality per 100,000 population members (CMR-suicide) 30–69 years of age, disaggregated by age/gender/employment status (employed/unemployed).

## 2. Materials and Methods

### 2.1. Ethics

This study conforms to the STROBE (Strengthening the Reporting of Observational Studies in Epidemiology) guidelines. The funding source contributed to shaping the research questions and supported data interpretation, but was not involved in the model construction, parameter selection, or methodological design.

### 2.2. Data Sources

Monthly suicide counts from January 2009 to December 2024, stratified by age (10-year intervals), gender, and employment status (unemployed and employed), were obtained from the “Basic Data on Suicide in Region” (BDSR), published by the Ministry of Health, Labor and Welfare (MHLW) [36,61,62]. Populations disaggregated by gender, age, and employment status were sourced from two official databases: the “Surveys of Population, Population Change, and the Number of Households based on the Basic Resident Registration” by the Ministry of Internal Affairs and Communications (MIAC) and the “Labor Force Survey” by MHLW [63,64]. Annual employment figures for individuals with physical, intellectual, and psychiatric disabilities were retrieved from the “Survey on the Actual Situation of Disabled Persons” conducted by MHLW [65]. Monthly crude suicide mortality rates per 100,000 population members (CMR-suicides), stratified by gender, age, and employment status, were calculated by dividing the monthly suicide counts by the corresponding population size for each subgroup. Finally, these monthly suicide mortalities were then annualized to reflect 365-day equivalents [35,37,38]. In Japan, over 60% of individuals aged 60–69 are employed, and a substantial proportion of those aged 20–29 are students who also engage in part-time work [64,66,67]. Therefore, this study focused on individuals aged 30–69 years as the primary target generation.

Monthly indices reflecting economic policy uncertainty (EPU) and government management instability (AENROP) were obtained from the database of the Research Institute of Economy, Trade and Industry (RIETI), affiliated with the Ministry of Economy, Trade and Industry [68]. The EPU index, widely recognized and available for Japan and 22 other countries, was originally developed by Baker and colleagues [69]. It integrates three components: the frequency of policy-related terms in major newspapers (via text mining), the number of federal tax code provisions, and the degree of disagreement among economic forecasters [69]. Japan’s EPU index specifically draws from four leading national newspapers—*Yomiuri*, *Asahi*, *Mainichi*, and *Nikkei*—to quantify domestic policy-related uncertainty [68]. The AENROP index was constructed by RIETI to measure instability in government management. It is based on monthly political party support ratings derived from public opinion polls conducted by news agencies (Jiji Press, Kyodo News), newspapers (*Yomiuri*, *Asahi*, *Mainichi*, *Nikkei*), and television networks (NHK, NNN, JNN) [68]. In both indices, higher values reflect elevated levels of uncertainty in economic policy and political governance, respectively [68,69].

Composite indices (CIs) of economic conditions were developed by the Economic and Social Research Institute (ESRI) under the Cabinet Office to assess Japan’s current economic status and forecast future trends [24].The CI framework comprises three distinct components: the leading index (leadCI), which typically precedes actual economic movements by several months; the coincident index (coinCI), which closely tracks the contemporaneous state of the economy; and the lagging index (lagCI), which reflects economic changes with a delay of several months [24,70]. These indices serve as critical tools for informing the formulation and revision of economic policies, as well as for guiding the preparation of the national budget by the Japanese government [24,70].

No missing data were observed in the dataset.

### 2.3. Statistical Analyses

Temporal fluctuations of CMR-suicides between 2009 and 2024 were examined using joinpoint regression analysis (JPRA) and interrupted time series analysis (ITSA). JPRA was conducted using the Joinpoint Regression Program (version 5.3.0; National Cancer Institute, Bethesda, MD, USA) [60,71], while ITSA was performed with Stata version 17 for Windows (StataCorp, College Station, TX, USA) [72,73,74]. JPRA applies a segmented regression approach to identify statistically significant inflection points where temporal trends shift. The model selects the simplest joinpoint configuration permitted by the data, thereby capturing meaningful changes in trend trajectories [71,75,76]. Selecting the model (the number of joinpoints) was accomplished by using the weighted Bayesian Information Criterion (weighted BIC).

Joinpoint regression analysis (JPRA) is a robust statistical technique for identifying inflection points—referred to as joinpoints—where long-term trends undergo significant changes [60,71,75]. The method partitions a continuous time series into multiple linear segments by minimizing the sum of squared residuals between observed values and fitted estimates, thereby optimizing the model fit [60,71]. JPRA is particularly effective for detecting unknown structural changes, such as transformed trends or discontinuities, within temporal datasets. Extensive methodological guides and statistical frameworks have been published to support its application [71,75]. While JPRA offers flexibility in analyzing joinpoints across extended observation periods—such as in suicide mortality trends—it tends to be conservative in identifying abrupt or transient fluctuations. This limitation may reduce its sensitivity to short-term discontinuities or sharply transformed trends [60,76].

Interrupted time series analysis (ITSA) is a widely utilized method for evaluating the effects of targeted interventions on temporal trends and structural discontinuities occurring around the intervention period [72,73,77]. ITSA offers considerable methodological flexibility, allowing for the incorporation of parametric and nonparametric regression models, seasonal adjustments, and panel data structures [72,73,77]. Leveraging these strengths, numerous studies have applied ITSA to assess the impact of the COVID-19 pandemic on fluctuations in suicide death rates (SDRs) and crude suicide mortality rates (CMR-suicides) [32,78,79]. Unlike JPRA, ITSA does not identify unknown fluctuations other than predefined intervention points. However, it demonstrates superior statistical power in detecting abrupt changes or discontinuities associated with known interventions [60,80,81].

Temporal causality from the indices of CI, EPU, and AENROP and the number of employed individuals with physical, intellectual, and psychiatric disabilities in relation to CMR-suicides from 2009 to 2024 were analyzed by vector-autoregressive analysis, with Granger causality and robust standard errors (VAR) [74,82,83], using Gretl for Windows (ver. 2025a) [84].

## 3. Results

### 3.1. Fluctuation of CMR-Suicides Among Overall-Ages Disaggregated by Gender and Employment Status

**Figure 2:**
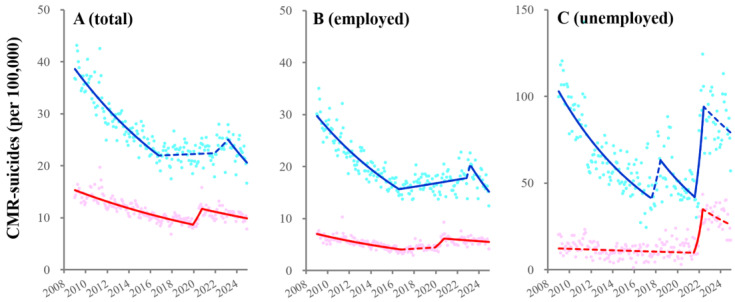
Fluctuations of CMR-suicides of total, employed and unemployed males and females of overall-ages from 2009–2024 using JPRA. Fluctuations of CMR-suicides among total (**A**), employed (**B**), and unemployed (**C**) males and females of overall-ages from January/2009 to December/2024 in Japan using JPRA. Ordinate and abscissa indicate the annualized monthly CMR-suicides per 100,000 population members and calendar years, respectively. Circles indicate the observed monthly CMR-suicides. Solid and dotted lines indicate the significant and non-significant trends of CMR-suicides, respectively. Blue and red indicate the data of males and females, respectively.

CMR-suicides among total males of overall-ages turned from decreasing to unchanging in 2016, followed by transiently increasing in 2022 and decreasing after the pandemic resolution, whereas those of females consistently decreased before the COVID-19 pandemic (until 2020) but sharply increased with the pandemic outbreak, followed by decreasing (Figure 2, Table S1 and Figure S1).

CMR-suicides among employed males of overall-ages turned from decreasing to increasing in 2016, followed by transiently increasing in 2022 and subsequently decreasing after the pandemic resolution (Figure 2, Table S1 and Figure S1). CMR-suicides among employed females of overall-ages turned from decreasing to unchanging in 2016, followed by sharply increasing with the pandemic outbreak and then weakly decreasing (Figure 2 and Figure S1).

Fluctuations of CMR-suicides among unemployed males of overall-ages indicated four joinpoints from 2009 to 2024: in 2016 (from decreasing to non-significantly/sharply increasing), in 2018 (to decreasing), in 2022 (to sharply increasing), and in 2023 (the pandemic resolution: maintaining high level) (Figure 2 and Table S1). CMR-suicides among unemployed females of overall-ages turned from unchanging to sharply increasing in 2022, followed by maintaining a high level (Figure 2 and Table S1).

### 3.2. Fluctuation of CMR-Suicides Disaggregated by Gender and Age

**Figure 3:**
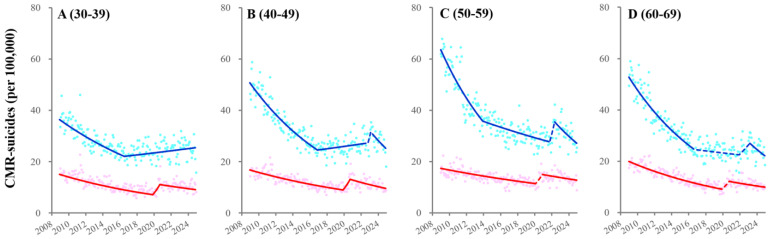
Fluctuations of CMR-suicides among total working-age males and females from 2009 to 2024 using JPRA. Fluctuations of CMR-suicides among 30–39- (**A**), 40–49- (**B**), 50–59- (**C**), and 60–69-years-of-age (**D**) males and females from January/2009 to December/2024 in Japan using JPRA. Ordinate and abscissa indicate the annualized monthly CMR-suicides (per 100,000 population members) and calendar years, respectively. Circles indicate the observed monthly CMR-suicides. Solid and dotted lines indicate the significant and non-significant trends of CMR-suicides, respectively. Blue and red indicate the data of males and females, respectively.

CMR-suicides among 30–39 males turned from decreasing to increasing in 2016, whereas CMR-suicides among 40–49 males turned from decreasing to increasing in 2016, followed by transiently increasing in 2022 and subsequently decreasing after the pandemic resolution (Figure 3, Table S2 and Figure S2). CMR-suicides among 30–49 females consistently decreased before the pandemic, followed by significantly/sharply increasing with the pandemic outbreak and subsequently decreasing (Figure 3 and Table S2). Decreasing trends of CMR-suicides among 50–69 males attenuated in 2016, followed by transiently increasing in 2022 and decreasing after the pandemic resolution (Figure 3, Table S2 and Figure S2). CMR-suicides among 50–69 females consistently decreased before the pandemic, followed by non-significantly/sharply increasing with the pandemic outbreak and subsequently decreasing (Figure 3 and Table S2).

### 3.3. Fluctuation of Employed and Unemployed CMR-Suicides Disaggregated by Gender and Age

**Figure 4:**
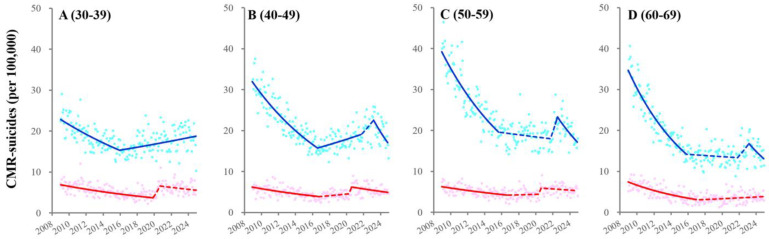
Fluctuations of CMR-suicides among employed males and females from 2009 to 2024 using JPRA. Fluctuations of CMR-suicides among 30–39- (**A**), 40–49- (**B**), 50–59- (**C**), and 60–69-years-of-age (**D**) employed males and females from January/2009 to December/2024 in Japan using JPRA. Ordinate and abscissa indicate the annualized monthly CMR-suicides (per 100,000 population members) and calendar years, respectively. Circles indicate the observed monthly CMR-suicides. Solid and dotted lines indicate the significant and non-significant trends of CMR-suicides, respectively. Blue and red indicate the data of males and females, respectively.

Temporal fluctuations of CMR-suicides among employed males and females disaggregated by age indicated similar patterns of totals corresponding to the same ages (Figure 4, Figures S3 and S4). Exceptionally, CMR-suicides among 60–69 employed females did not respond to the pandemic outbreak, and decreasing trends of CMR-suicides among 30–69 employed females attenuated in 2016 (Figure 4, Table S3 and Figure S4).

**Figure 5:**
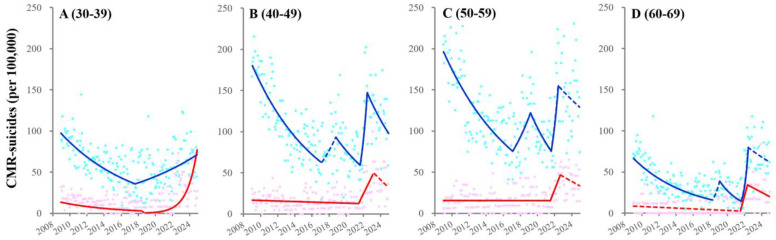
Fluctuations of CMR-suicides among unemployed males and females from 2009 to 2024. Fluctuations of CMR-suicides among 30−39- (**A**), 40−49- (**B**), 50−59- (**C**), and 60−69-years-of-age (**D**) unemployed males and females from January/2009 to December/2024 in Japan using JPRA. Ordinate and abscissa indicate the annualized monthly CMR-suicides (per 100,000 population members) and calendar years, respectively. Circles indicate the observed monthly CMR-suicides. Solid and dotted lines indicate the significant and non-significant trends of CMR-suicides, respectively. Blue and red indicate the data of males and females, respectively.

In the 30–39 generation, CMR-suicides among unemployed males and females turned from decreasing to increasing in 2016 and 2018, respectively (Figure 5 and Table S4). Fluctuations of CMR-suicides among 40–69 unemployed males indicated four joinpoints: in 2016 (from decreasing to increasing), in 2018 (to decreasing), in 2022 (to increasing), and in 2023 (the pandemic resolution: to decreasing or unchanging) (Figure 5 and Table S4). CMR-suicides among 40–59 unemployed females turned from decreasing to sharply increasing in 2022, followed by maintaining a high level (Figure 5 and Table S4). CMR-suicides among 60–69 unemployed females turned from unchanging to sharply increasing in 2022, followed by decreasing with the pandemic resolution (Figure 5 and Table S4).

### 3.4. Impacts of Economic Condition, Uncertainty Indices, and Numbers of Employed People with Disabilities on Employed and Unemployed CMR-Suicides Disaggregated by Gender and Age

All CMR-suicides among 30−69 employed males/females negatively related to lagCI, but were unrelated to leadCI and coinCI (Table 1), meaning that CMR-suicides among employed individuals increased approximately several months after their actual economic status reduced. On the contrary, CMR-suicides among 30−69 unemployed males and females did not relate to any CI indices (Table 1). Therefore, an economic downturn is a suicide risk factor for employed individuals, but unemployed individuals are less sensitive to that economic condition.

AENROP positively related to CMR-suicides among 30–69 employed and unemployed males. On the contrary, 30–39 employed females positively related to AENROP, but 40–69 did not relate to AENROP (Table 2). However, CMR-suicides among all unemployed individuals did not relate to EPU (Table 2). The positive impacts of AENROP on employed males CMR-suicides were more pronounced than those of lagCI, whereas the protective impacts of lagCI on employed females CMR-suicides were greater than those of AENROP (Table 3).

Increasing employees with psychiatric disabilities positively related to CMR-suicides among 30–39 employed and unemployed males and females and 40–69 unemployed males (Table 4). Conversely, increasing employees with intellectual disabilities negatively related to CMR-suicides among 30–39 employed males, 30–39 unemployed males/females, and 40–59 unemployed males (Table 4). Increasing employees with physical disabilities also negatively related to CMR-suicides among 30–39 employed/unemployed males (Table 4). Therefore, increasing employment rates of individuals with psychiatric disabilities contributed to increasing CMR-suicides among unemployed males, 30–39 employed males, and employed/unemployed females.

## 4. Discussion

This study demonstrated the complicated fluctuations and their causalities for CMR-suicides among 30–69 employed/unemployed males/females from 2009 to 2024 using time series analyses (JPRA and ITSA) and causality analysis (VAR). All CMR-suicides consistently decreased from 2009 to 2016, but subsequently, five joinpoints were detected: in 2016 (from decreasing to increasing in employed males/females and unemployed males), in 2018 (to decreasing in unemployed males), in 2020 (to sharply increasing in employed females), in 2022 (to sharply increasing in employed males and unemployed males/females), and in 2023 (to decreasing in employed males or maintaining a high level in unemployed males/females). Notably, fluctuations of CMR-suicides among 30–69 employed males/females were involved in fluctuations of overall CMR-suicides among the corresponding gender/age, but CMR-suicides among unemployed individuals had extremely minor impacts on the overall CMR-suicides. However, even if the impacts of suicides among unemployed individuals on the overall suicide mortality are minor, the increasing suicides among unemployed individuals are major public health concerns in current Japan, since CMR-suicides among unemployed males and females increased over 2 times (ranged 2.1–5.6) and 3 times (ranged 3.1–14.2) in the 2020s. Additionally, unemployment is an established suicide risk, whereas this study demonstrated that CMR-suicides among unemployed individuals largely diverted, independent of economic status. Five joinpoints of CMR-suicides were detected, in which four joinpoints temporally related to political events, but only one (the COVID-19 pandemic outbreak) related to an economic downturn. The other four joinpoints temporally related to implementations of the “Act on Employment Promotion of Persons with Disabilities” in 2016, when employment of people with physical and intellectual disabilities was made mandatory (psychiatric disability was limited to being recommended); the “Work Style Reform Act” and “revised Act on Employment Promotion of Persons with Disabilities” in 2018, when the employment environment/welfare were improved and the employment of people with psychiatric disabilities was made mandatory; the “revision of economic supportive countermeasures against economic deterioration caused by COVID-19” in 2022, when governmental countermeasures for economic deterioration induced by the COVID-19 pandemic, such as the “Sustainability Benefit”, were revised and reduced financial supports; and the pandemic resolution in 2023.

### 4.1. Impacts of Economic Status

In contrast to the prevailing statistical consensus on suicide trends, only the CMR-suicides among employed females increased with the COVID-19 pandemic outbreak, whereas the CMR-suicides among employed males and unemployed individuals of both genders were unrelated to the pandemic. On the contrary, in 2022, CMR-suicides among 40–69 employed males and unemployed males/females sharply increased, whereas trends of CMR-suicides among 30–69 employed females and 30–39 unemployed males/females remained unchanged. After the pandemic resolution, increased CMR-suicides among unemployed 40–69 males/females maintained a high level, whereas those among 40–69 employed males turned to decreasing. These complicated fluctuations can be plausibly explained by the responses to indices of uncertainty and CI. CMR-suicides among employed males/females were unrelated to EPU, but positively and protectively related to AENROP and lagCI, respectively. The impacts of lagCI on CMR-suicides among 30–59 employed females were more pronounced than those of AENROP, whereas the impacts of AENROP on CMR-suicides among employed males and 60–69 employed females were greater than those of lagCI. Therefore, the majority of Japanese employed individuals are sensitive to economic activity that directly affects their lives but are not interested in or affected by predictive economic information from mass media; however, employed 30–69 males and 60–69 females, who showed unchanged CMR-suicides with the outbreak, had higher sensitivity to AENROP (government political information) compared to economic status. Indeed, in 2022, CMR-suicides among 40–69 employed males weakly increased with the implementation of the “Business Revitalization Support Fund”, which shifted government expenditures from financial support for small/medium-sized enterprises to maintain employment to support for restarting business activities. Recent studies reported that the impacts of EPU and AENROP on suicides were greater compared to the unemployment rates in high-income countries, including the United States, the United Kingdom, and Japan, possibly due to generous unemployment insurance programs [50,51,52,57,85]. The discrepancies in the response between employed and unemployed CMR-suicides to lagCI can be interpreted as it being rational that unemployed individuals, who already lost their jobs, are more sensitive to government information that manages employment opportunities in generous high-welfare countries than to information about the risk for losing their jobs in the future. CMR-suicides among unemployed males/females sharply/drastically increased in 2022, subsequently remaining high after the pandemic resolution. The sharply increasing CMR-suicides among unemployed individuals observed during and after the COVID-19 pandemic may be more plausibly attributed to the intensification of economic hardship resulting from the prolonged crisis rather than to the effects of the “Business Revitalization Support Fund”. Notably, the number of beneficiaries of the “Housing Security Benefits”—a government program providing rental assistance to unemployed individuals experiencing financial distress—rose dramatically, increasing over 30-fold, from 3972 cases in 2019 to 134,946 cases in 2020 [86]. In response to the pandemic, the coverage period of this program was extended from three to twelve months, beginning in January 2021. However, in 2022, many unemployed individuals were at risk of losing not only their unemployment benefits but also their housing support, as temporary COVID-19-related assistance measures began to phase out. The termination of key economic support programs, including the “COVID-19 Leave Support Payments and Subsidies”, in May 2023—marking the formal resolution of the pandemic—may have played a critical role in sustaining elevated levels of CMR-suicides among unemployed individuals from 2022 onward [57,86]. Taken together, these findings suggest that the extreme economic hardship faced by unemployed individuals, particularly in the context of diminishing governmental financial supports or social safety systems, may plausibly explain both the sharply increasing and the sustained high levels of CMR-suicides among unemployed males/females during the late phase and post-pandemic periods.

### 4.2. Impacts of Promotion of Employment for Individuals with Disabilities

In Japan, the “Act on Promotion of Employment of Persons with Disabilities” implemented in 2016 and 2018 made the employment of individuals with disabilities mandatory. Therefore, the implementation of these acts probably plays an important role in the increasing or attenuated decreasing trends of CMR-suicides among 30–69 employed/unemployed males and 30–39 employed/unemployed females, in which CMR-suicides are positively related to the increasing employment of individuals with psychiatric disabilities. The “Work Style Reform Act” in 2018 improved the employment environment/welfare, leading to also promoting the employment of individuals with disabilities. The majority of individuals with psychiatric disabilities who adopt the employment programs for people with disabilities are individuals suffering from schizophrenia and mood disorders in Japan [65]. The continuity of employment of individuals with psychiatric disorders is short [87,88,89]; especially in Japan, over 50% of employed individuals with psychiatric disorders are unable to continue working within a year [65], resulting in them being registered as unemployed and restarting job seeking. Considering these current situations, individuals with psychiatric disorders, who are at high risk for suicide, have increasingly been registered in not only the employed but also the unemployed categories, which may have contributed to the apparent increasing suicides among both employed and unemployed individuals in the statistics. The majority of certifications/diagnoses of intellectual and physical disabilities is almost completed before school age, and opportunities for education/training to acquire employment skills for people who have a disability are provided in special needs schools [90,91]. The highest employment rate for individuals with psychiatric disabilities was in the 25–39 group [65]. The median age of diagnosis of these psychiatric disorders is approximately 30 years [92,93]. Individuals with psychiatric disorders can participate in employment programs for people with disabilities within one year, at the earliest, after being diagnosed with a psychiatric disorder [65]. Therefore, concern remains regarding the lack of education/training for individuals with psychiatric disabilities to acquire various skills necessary to stay in the workforce for the long term [91,94]. Indeed, the provision of employment skills for people with disabilities is provided in the governmental “Hello Training” program, but the annual number of individuals using this program has been approximately 5000, which was extremely less than the 150,000 employed individuals with psychiatric disabilities employed in 2024. The “Act on Promotion of Employment of Persons with Disabilities” was amended again in 2023 to provide financial support for environmental improvement in order to promote the provision of employment assistance according to the characteristics of the disability of individuals with disabilities. Therefore, the findings in this study support the basis/rationality of improving the quality of employment for individuals with disabilities, which is currently being promoted by the government, from the perspective of suicide statistics.

Unemployment has been considered as one of the impactable suicide risks; however, the actual suicide risk for unemployed individuals remains to be clarified [3,95]. This study demonstrated two candidate causalities underlying drastically increasing CMR-suicides among unemployed individuals during the 2020s in Japan. Suicides among unemployed individuals are predominantly affected by the economic hardship associated with the government unemployment insurance programs (economic factors that directly affect their lives) rather than by socioeconomic deterioration. Currently, social support for individuals with psychiatric disabilities is considered to have great potential for suicide prevention and improving quality of life; so, employment programs for people with disabilities have been promoted, resulting in increasing employed individuals with psychiatric disorders using this program [65,96]. However, this study suggests the need to promote employment support and environmental improvements according to the characteristics of the disabilities of individuals.

### 4.3. Limitations

There were several limitations in this study. The indices of EPU and AENROP for Japan are promoted using text mining techniques from major mass media. Currently, social media has emerged as a powerful force in shaping public discourse on social issues, with an impact comparable to that of conventional mass media [97]. However, the EPU and AENROP indices do not reflect social media content. Therefore, the impacts of the EPU and AENROP indices detected in this study may not fully capture the dynamics of recent sociopolitical environments. Additionally, Granger causality analysis in the VAR framework can indicate the possibility of temporal precedence and predictive relevance but does not confirm actual structural or theoretical causalities. Therefore, the findings should be interpreted as hypothesis-generating rather than conclusive evidence of causal pathways [98].

## 5. Conclusions

This study demonstrated the complicated fluctuations of CMR-suicides, disaggregated by age, gender, and social standing (employed/unemployed), and their causalities from 2009 to 2024. Fluctuations in CMR-suicides across Japan were largely reflected by CMR-suicides among employed males and females. Importantly, the sharply increasing CMR-suicides among employed females at the COVID-19 pandemic outbreak was dependent on the predominant sensitivity to lagCI (economic expanding) rather than AENROP (government information); conversely, the fact of no changing CMR-suicides among employed males at the outbreak is possibly due to the dominant sensitivity to government information on countermeasures rather than the deterioration of the economic situation. Suicides among unemployed individuals have a very small impact on overall suicide mortality, whereas CMR-suicides among unemployed males and females increased in 2022, without changing at the COVID-19 pandemic outbreak, over 2-fold (ranged 2.1–5.6) and 3-fold (ranged 3.1–14.2), respectively, probably caused by prolonged economic hardship. Unexpectedly, before the pandemic, Japan experienced increasing employment of individuals with psychiatric disabilities due to implementation of the “Act on Employment Promotion of Persons with Disabilities” in 2016. Although implementation of the “work style reform Act” in 2018 suppressed increasing CMR-suicides among those 40–69, CMR-suicides among 30–39 unemployed males persisted in increasing. These findings in this study further emphasize the need for further enhancement of the welfare of individuals with psychiatric disabilities in workplaces, which is currently being promoted by the government. Furthermore, this study revealed that suicides are temporally and fundamentally complicated phenomena, so it is necessary to continue monitoring them, not only to apply suicide risk factors that have been established traditionally, but also to note the possibility that unknown suicide risk factors are constantly developing due to social structure transformation and welfare/economic policies.

## Figures and Tables

**Table 1:** Temporal causality from CI indices for CMR-suicides among employed and unemployed males/females from 2009 to 2024 using VAR.

Employed Males										
Age	R^2^	F	*p*			β	SE	T	*p*	
30−39	0.324	22.3	<0.001	**	leadCI	−0.733	0.441	−1.662	0.098	
					coinCI	−0.056	0.092	−0.608	0.544	
					lagCI	−1.419	0.421	−3.372	0.001	**
40−49	0.639	61.6	<0.001	**	leadCI	−1.151	0.889	−1.294	0.197	
					coinCI	0.274	0.991	0.276	0.783	
					lagCI	−2.893	1.163	−2.488	0.014	*
50−59	0.768	107.9	<0.001	**	leadCI	−1.673	1.025	−1.633	0.104	
					coinCI	1.308	1.136	1.151	0.251	
					lagCI	−2.987	1.401	−2.131	0.034	*
60−69	0.824	223.3	<0.001	**	leadCI	−1.072	0.866	−1.238	0.217	
					coinCI	0.661	0.889	0.743	0.458	
					lagCI	−2.065	1.020	−2.024	0.044	*
**Unemployed Males**										
**Age**	**R^2^**	**F**	***p***			**β**	**SE**	**T**	***p***	
30−39	0.242	16.7	<0.001	**	leadCI	−3.315	6.212	−0.534	0.594	
					coinCI	0.005	7.144	0.001	0.999	
					lagCI	−9.866	6.527	−1.512	0.132	
40−49	0.472	50.4	<0.001	**	leadCI	−6.230	8.928	−0.698	0.486	
					coinCI	0.770	9.012	0.085	0.932	
					lagCI	−12.094	9.261	−1.306	0.193	
50−59	0.427	30.1	<0.001	**	leadCI	1.629	10.785	0.151	0.880	
					coinCI	−8.655	11.311	−0.765	0.445	
					lagCI	−2.812	10.970	−0.256	0.798	
60−69	0.370	21.3	<0.001	**	leadCI	3.287	6.357	0.517	0.606	
					coinCI	−6.601	6.784	−0.973	0.332	
					lagCI	2.978	6.164	0.483	0.630	
**Employed Females**										
**Age**	**R^2^**	**F**	***p***			**β**	**SE**	**T**	***p***	
30−39	0.274	18.0	<0.001	**	leadCI	−0.070	0.156	−0.447	0.655	
					coinCI	−0.044	0.041	−1.064	0.289	
					lagCI	−0.996	0.207	−4.806	<0.001	**
40−49	0.186	10.8	<0.001	**	leadCI	0.073	0.161	0.450	0.654	
					coinCI	−0.027	0.039	−0.686	0.494	
					lagCI	−0.810	0.174	−4.651	<0.001	**
50−59	0.146	8.8	<0.001	**	leadCI	0.030	0.180	0.166	0.869	
					coinCI	−0.053	0.035	−1.507	0.133	
					lagCI	−0.686	0.169	−4.058	<0.001	**
60−69	0.370	27.6	<0.001	**	leadCI	−0.428	0.240	−1.785	0.076	
					coinCI	0.014	0.044	0.308	0.759	
					lagCI	−1.498	0.230	−6.510	<0.001	**
**Unemployed Females**										
Age	**R^2^**	**F**	***p***			**β**	**SE**	**T**	***p***	
30−39	0.243	9.9	<0.001	**	leadCI	2.091	3.708	0.564	0.574	
					coinCI	−2.619	3.852	−0.680	0.497	
					lagCI	1.753	3.452	0.508	0.612	
40−49	0.181	9.0	<0.001	**	leadCI	5.443	3.570	1.525	0.129	
					coinCI	−6.407	3.700	−1.731	0.085	
					lagCI	6.718	3.586	1.874	0.063	
50−59	0.100	5.0	0.001	**	leadCI	6.115	3.843	1.591	0.113	
					coinCI	−5.457	4.012	−1.360	0.175	
					lagCI	6.410	.3.781	1.696	0.092	
60−69	0.127	5.6	<0.001	**	leadCI	3.586	3.545	1.011	0.313	
					coinCI	−2.804	3.507	−0.800	0.425	
					lagCI	1.671	3.582	0.467	0.641	

Composite indices (CIs): leading index (leadCI), which moves ahead (approximately several months) of the economy; coincident index (coinCI), which moves almost in sync with the actual economy; and lagging index (lagCI), which moves behind (approximately several months) the actual economy. R^2^: coefficient of determination, F: F-value, SE: standard error, β: coefficient value, T: T-value. * *p* < 0.05, ** *p* < 0.01.

**Table 2:** Temporal causality from indices of EPU and AENROP for CMR-suicides among employed and unemployed males/females from 2009 to 2024 using VAR.

Employed Males										
Age	R^2^	F	*p*			β	SE	T	*p*	
30–39	0.273	23.3	<0.001	**	AENROP	0.160	0.064	2.486	0.014	*
					EPU	−0.029	0.075	−0.387	0.700	
40–49	0.609	70.3	<0.001	**	AENROP	0.287	0.080	3.589	<0.001	**
					EPU	−0.022	0.083	−0.271	0.787	
50–59	0.762	125.6	<0.001	**	AENROP	0.202	0.061	3.312	0.001	**
					EPU	−0.059	0.078	−0.750	0.454	
60–69	0.809	228.7	<0.001	**	AENROP	0.146	0.070	2.070	0.040	*
					EPU	−0.051	0.060	−0.855	0.394	
**Unemployed Males**										
**Age**	**R^2^**	**F**	***p***			**β**	**SE**	**T**	***p***	
30–39	0.233	19.3	<0.001	**	AENROP	1.576	0.453	3.478	0.001	**
					EPU	−0.275	0.650	−0.422	0.673	
40–49	0.469	59.7	<0.001	**	AENROP	2.044	0.787	2.599	0.010	*
					EPU	−0.303	0.757	−0.401	0.689	
50–59	0.762	125.6	<0.001	**	AENROP	0.202	0.061	3.312	0.001	**
					EPU	−0.059	0.078	−0.750	0.454	
60–69	0.387	26.3	<0.001	**	AENROP	1.123	0.380	2.957	0.004	**
					EPU	−0.562	0.381	−1.474	0.142	
**Employed Females**										
**Age**	**R^2^**	**F**	***p***			**β**	**SE**	**T**	***p***	
30–39	0.196	21.3	<0.001	**	AENROP	0.007	0.004	1.990	0.048	*
					EPU	0.001	0.003	0.214	0.831	
40–49	0.113	10.5	<0.001	**	AENROP	0.057	0.028	2.063	0.041	*
					EPU	−0.016	0.030	−0.545	0.587	
50–59	0.096	7.4	<0.001	**	AENROP	0.058	0.025	2.310	0.022	*
					EPU	−0.036	0.032	−1.105	0.270	
60–69	0.256	20.5	<0.001	**	AENROP	0.147	0.031	4.673	<0.001	**
					EPU	0.055	0.040	1.363	0.175	
**Unemployed Females**										
**Age**	**R^2^**	**F**	***p***			**β**	**SE**	**T**	***p***	
30–39	0.265	14.3	<0.001	**	AENROP	0.053	0.022	2.460	0.015	*
					EPU	−0.038	0.029	−1.321	0.188	
40–49	0.190	12.0	<0.001	**	AENROP	0.403	0.236	1.710	0.089	
					EPU	−0.564	0.324	−1.740	0.084	
50–59	0.107	6.9	<0.001	**	AENROP	0.340	0.257	1.325	0.187	
					EPU	−0.694	0.395	−1.759	0.080	
60–69	0.133	9.8	<0.001	**	AENROP	0.345	0.263	1.311	0.191	
					EPU	−0.067	0.337	−0.197	0.844	

EPU: economic policy uncertainty, AENROP: government management instability, R^2^: coefficient of determination, F: F-value, SE: standard error, β: coefficient value, T: T-value. * *p* < 0.05, ** *p* < 0.01.

**Table 3:** Temporal causality from indices of AENROP and lagCI for CMR-suicides among employed males and females from 2009 to 2024 using VAR.

Employed Males										
Age	R2	F	*p*			β	SE	T	*p*	
30–39	0.303	26.0	<0.001	**	AENROP	0.139	0.061	2.302	0.023	*
					lagCI	−0.269	0.101	−2.662	0.008	**
40–49	0.628	79.7	<0.001	**	AENROP	0.277	0.076	3.672	<0.001	**
					lagCI	−0.334	0.121	−2.757	0.006	**
50–59	0.766	148.9	<0.001	**	AENROP	0.190	0.055	3.457	0.001	**
					lagCI	−0.219	0.119	−1.840	0.067	
60–69	0.811	240.6	<0.001	**	AENROP	0.142	0.066	2.148	0.033	*
					lagCI	−0.157	0.100	−1.565	0.119	
**Employed Females**										
**Age**	**R2**	**F**	***p***			**β**	**SE**	**T**	***p***	
30–39	0.270	22.9	<0.001	**	AENROP	0.005	0.040	0.112	0.911	
					lagCI	−0.935	0.220	−4.260	<0.001	**
40–49	0.188	14.4	<0.001	**	AENROP	0.005	0.028	0.184	0.854	
					lagCI	−0.735	0.169	−4.358	<0.001	**
50–59	0.140	11.6	<0.001	**	AENROP	0.015	0.025	0.588	0.557	
					lagCI	−0.552	0.175	−3.158	0.002	**
60–69	0.386	40.2	<0.001	**	AENROP	0.098	0.028	3.458	0.001	**
					lagCI	−1.375	0.211	−6.527	<0.001	**

EPU: economic policy uncertainty, AENROP: government management instability, R^2^: coefficient of determination, F: F-value, SE: standard error, β: coefficient value, T: T-value. * *p* < 0.05, ** *p* < 0.01.

**Table 4:** Temporal causality from employment rates of people with physical, intellectual, and psychiatric disabilities for CMR-suicides among employed and unemployed males/females from 2009 to 2024 using VAR.

Employed Males										
Age	R2	F	*p*			β	SE	T	*p*	
30–39	0.744	28.6	0.000	**	Physical	0.003	0.005	0.707	0.496	
					Intellectual	−0.081	0.035	−2.349	0.041	*
					Psychiatric	0.121	0.046	2.632	0.025	*
40–49	0.819	5.6	0.013	*	Physical	−0.200	0.220	−0.909	0.385	
					Intellectual	0.902	1.052	0.857	0.412	
					Psychiatric	−0.264	0.359	−0.737	0.478	
50–59	0.549	27.2	0.000	**	Physical	0.015	0.019	0.769	0.460	
					Intellectual	−0.362	0.573	−0.632	0.542	
					Psychiatric	0.234	0.350	0.670	0.518	
60–69	0.767	42.2	0.000	**	Physical	0.121	0.103	1.178	0.266	
					Intellectual	−2.923	3.453	−0.846	0.417	
					Psychiatric	−2.566	2.160	−1.188	0.262	
**Unemployed Males**										
**Age**	**R2**	**F**	***p***			**β**	**SE**	**T**	***p***	
30–39	0.607	60.0	0.000	**	Physical	−0.079	0.022	−3.547	0.005	**
					Intellectual	−0.632	0.121	−5.236	0.000	**
					Psychiatric	0.409	0.090	4.539	0.001	**
40–49	0.366	28.3	0.000	**	Physical	−0.338	0.105	−3.229	0.009	**
					Intellectual	−1.192	0.366	−3.258	0.009	**
					Psychiatric	0.806	0.295	2.733	0.021	*
50–59	0.394	22.4	0.000	**	Physical	0.151	0.072	2.093	0.063	
					Intellectual	−2.178	0.812	−2.682	0.023	*
					Psychiatric	0.830	0.320	2.590	0.027	*
60–69	0.456	1.9	0.190		Physical	0.159	0.109	1.461	0.175	
					Intellectual	−2.170	1.043	−2.081	0.064	
					Psychiatric	0.697	0.249	2.803	0.019	*
**Employed Females**										
**Age**	**R2**	**F**	***p***			**β**	**SE**	**T**	***p***	
30–39	0.690	45.9	0.000	**	Physical	−0.002	0.002	−1.244	0.242	
					Intellectual	−0.060	0.028	−2.162	0.056	
					Psychiatric	0.071	0.031	2.265	0.047	*
40–49	0.790	10.7	0.001	**	Physical	−0.062	0.063	−0.983	0.349	
					Intellectual	0.291	0.336	0.866	0.407	
					Psychiatric	−0.083	0.116	−0.719	0.488	
50–59	0.651	7.1	0.005	**	Physical	0.003	0.003	0.988	0.347	
					Intellectual	−0.095	0.141	−0.673	0.516	
					Psychiatric	0.071	0.099	0.716	0.490	
60–69	0.789	167.2	0.000	**	Physical	0.028	0.021	1.296	0.224	
					Intellectual	−0.908	0.742	−1.223	0.249	
					Psychiatric	−0.547	0.436	−1.254	0.239	
**Unemployed Females**										
**Age**	**R2**	**F**	***p***			**β**	**SE**	**T**	***p***	
30–39	0.614	65.1	0.000	**	Physical	−0.018	0.019	−0.914	0.382	
					Intellectual	−0.328	0.103	−3.187	0.010	*
					Psychiatric	0.340	0.093	3.661	0.004	**
40–49	0.716	3.2	0.062		Physical	−0.306	0.146	−2.097	0.062	
					Intellectual	1.583	0.930	1.702	0.120	
					Psychiatric	−0.514	0.381	−1.347	0.208	
50–59	0.365	1.5	0.269		Physical	0.019	0.020	0.940	0.370	
					Intellectual	−0.275	0.408	−0.675	0.515	
					Psychiatric	0.231	0.288	0.801	0.442	
60–69	0.667	1.4	0.299		Physical	0.247	0.357	0.691	0.505	
					Intellectual	−8.901	13.050	−0.682	0.511	
					Psychiatric	−4.390	8.184	−0.536	0.603	

* *p* < 0.05, ** *p* < 0.01.

## Data Availability

The data presented in this study are available in Japanese national databases from the “Basic Data on Suicide in the Region”, https://www.e-stat.go.jp/en/statistics/00200531 (accessed on 1 July 2025); “Labor Force Survey”, https://www.e-stat.go.jp/en/statistics/00200531 (accessed on 1 July 2025); “Survey on the Actual Situation of Disabled Persons”, https://www.mhlw.go.jp/toukei/list/111-1.html (accessed on 1 July 2025), in MHLW; and “Surveys of Population, Population Change and the Number of Households based on the Basic Resident Registration”, https://www.e-stat.go.jp/en/statistics/00200241 (accessed on 1 July 2025), in e-Stat (Ministry of Internal Affairs and Communications). Indices of EPU and AENROP were published by the Research Institute of Economy, Trade, and Industry (RIETI) in the Ministry of Economy, Trade and Industry, https://www.rieti.go.jp/jp/database/policyuncertainty/index.html (accessed on 1 July 2025), and CI indices were obtained from the Economic and Social Research Institute (ESRI) in the Cabinet Office https://www.rieti.go.jp/jp/database/policyuncertainty/index.html (accessed on 1 July 2025).

## Supplementary Materials

The following supporting information can be downloaded at: https://www.mdpi.com/article/10.3390/ijerph22091315/s1, Supplementary Table S1: APC (±95%CI) of CMR-suicides among total, employed, and unemployed males and females of overall-ages from 2009 to 2024 using JPRA; Supplementary Table S2: APC (±95%CI) of CMR-suicides among total working-age males and females from 2009 to 2024 using JPRA; Supplementary Table S3: APC (±95%CI) of CMR-suicides among employed working-age males and females from 2009 to 2024 using JPRA; Supplementary Table S4: APC (±95%CI) of CMR-suicides among unemployed working-age males and females from 2009 to 2024 using JPRA; Supplementary Figure S1: Fluctuations of CMR-suicides among total and employed males and females of overall-ages from 2009 to 2024 using ITSA; Supplementary Figure S2: Fluctuations of CMR-suicides among 40–69 total males from 2009 to 2024 using ITSA; Supplementary Figure S3: Fluctuations of CMR-suicides among 40–69 employed males from 2009 to 2024 using ITSA; Supplementary Figure S4: Fluctuations of CMR-suicides among 30–59 employed females from 2009 to 2024 using ITSA.

## Abbreviations

The following abbreviations are used in this manuscript:

AENROP	government management instability
BDSR	Basic Data on Suicide in Region
CIs	composite indices
CMR	crude suicide mortality rate
coinCI	Coincident composite index
EPU	economic policy uncertainty
ESRI	Economic and Social Research Institute
ITSA	interrupted time series analysis
JPRA	joinpoint regression
lagCI	Lagging composite index
leadCI	Leading composite index
MIAC	Ministry of Internal Affairs and Communications
MHLW	Ministry of Health, Labor and Welfare
RIETI	Research Institute of Economy, Trade, and Industry
VAR	vector-autoregressive analysis with Granger causality and robust standard errors

## References

[1] Sinyor M., Silverman M., Pirkis J., Hawton K. (2024). The effect of economic downturn, financial hardship, unemployment, and relevant government responses on suicide. Lancet Public Health.

[2] Mathieu S., Treloar A., Hawgood J., Ross V., Kolves K. (2022). The Role of Unemployment, Financial Hardship, and Economic Recession on Suicidal Behaviors and Interventions to Mitigate Their Impact: A Review. Front. Public Health.

[3] Roelfs D.J., Shor E. (2023). Financial Stress, Unemployment, and Suicide—A Meta-Analysis. Crisis.

[4] Na P.J., Shin J., Kwak H.R., Lee J., Jester D.J., Bandara P., Kim J.Y., Moutier C.Y., Pietrzak R.H., Oquendo M.A. (2025). Social Determinants of Health and Suicide-Related Outcomes: A Review of Meta-Analyses. JAMA Psychiatry.

[5] Favril L., Yu R., Geddes J.R., Fazel S. (2023). Individual-level risk factors for suicide mortality in the general population: An umbrella review. Lancet Public Health.

[6] Silva M., Resurreccion D.M., Antunes A., Frasquilho D., Cardoso G. (2018). Impact of economic crises on mental health care: A systematic review. Epidemiol. Psychiatr. Sci..

[7] The Lancet Public Health (2024). A public health approach to suicide prevention. Lancet Public Health.

[8] Swinscow D. (1951). Some suicide statistics. Br. Med. J..

[9] Chang S.S., Gunnell D., Sterne J.A., Lu T.H., Cheng A.T. (2009). Was the economic crisis 1997–1998 responsible for rising suicide rates in East/Southeast Asia? A time-trend analysis for Japan, Hong Kong, South Korea, Taiwan, Singapore and Thailand. Soc. Sci. Med..

[10] Chang S.S., Stuckler D., Yip P., Gunnell D. (2013). Impact of 2008 global economic crisis on suicide: Time trend study in 54 countries. BMJ.

[11] Kato R., Okada M. (2019). Can Financial Support Reduce Suicide Mortality Rates?. Int. J. Environ. Res. Public Health.

[12] Jeon S.Y., Reither E.N., Masters R.K. (2016). A population-based analysis of increasing rates of suicide mortality in Japan and South Korea, 1985–2010. BMC Public Health.

[13] Russell R., Metraux D., Tohen M. (2017). Cultural influences on suicide in Japan. Psychiatry Clin. Neurosci..

[14] Chen Y.Y., Wu K.C., Yousuf S., Yip P.S. (2012). Suicide in Asia: Opportunities and challenges. Epidemiol. Rev..

[15] Kim S.Y., Kim M.H., Kawachi I., Cho Y. (2011). Comparative epidemiology of suicide in South Korea and Japan: Effects of age, gender and suicide methods. Crisis.

[16] Otsu A., Araki S., Sakai R., Yokoyama K., Scott Voorhees A. (2004). Effects of urbanization, economic development, and migration of workers on suicide mortality in Japan. Soc. Sci. Med..

[17] Stuckler D., Basu S., Suhrcke M., Coutts A., McKee M. (2011). Effects of the 2008 recession on health: A first look at European data. Lancet.

[18] Gili M., Roca M., Basu S., McKee M., Stuckler D. (2013). The mental health risks of economic crisis in Spain: Evidence from primary care centres, 2006 and 2010. Eur. J. Public Health.

[19] Barr B., Taylor-Robinson D., Scott-Samuel A., McKee M., Stuckler D. (2012). Suicides associated with the 2008-10 economic recession in England: Time trend analysis. BMJ.

[20] De Vogli R., Marmot M., Stuckler D. (2013). Strong evidence that the economic crisis caused a rise in suicides in Europe: The need for social protection. J. Epidemiol. Community Health.

[21] Huikari S., Miettunen J., Korhonen M. (2019). Economic crises and suicides between 1970 and 2011: Time trend study in 21 developed countries. J. Epidemiol. Community Health.

[22] Phillips J.A., Nugent C.N. (2014). Suicide and the Great Recession of 2007–2009: The role of economic factors in the 50 U.S. states. Soc. Sci. Med..

[23] Matsubayashi T., Sekijima K., Ueda M. (2020). Government spending, recession, and suicide: Evidence from Japan. BMC Public Health.

[24] ESRI Indexes of Business Conditions. https://www.esri.cao.go.jp/en/stat/di/di-e.html.

[25] Takeshima T., Yamauchi T., Inagaki M., Kodaka M., Matsumoto T., Kawano K., Katsumata Y., Fujimori M., Hisanaga A., Takahashi Y. (2015). Suicide prevention strategies in Japan: A 15-year review (1998–2013). J. Public Health Policy.

[26] Okada M., Hasegawa T., Kato R., Shiroyama T. (2020). Analysing regional unemployment rates, GDP per capita and financial support for regional suicide prevention programme on suicide mortality in Japan using governmental statistical data. BMJ Open.

[27] Hasegawa T., Matsumoto R., Yamamoto Y., Okada M. (2021). Analysing effects of financial support for regional suicide prevention programmes on methods of suicide completion in Japan between 2009 and 2018 using governmental statistical data. BMJ Open.

[28] McKibbin W., Vines D. (2020). Global macroeconomic cooperation in response to the COVID-19 pandemic: A roadmap for the G20 and the IMF. Oxf. Rev. Econ. Policy.

[29] Arya V., Page A., Spittal M.J., Dandona R., Vijayakumar L., Munasinghe S., John A., Gunnell D., Pirkis J., Armstrong G. (2022). Suicide in India during the first year of the COVID-19 pandemic. J. Affect. Disord..

[30] Menon V., Cherian A.V., Vijayakumar L. (2021). Rising incidence and changing demographics of suicide in India: Time to recalibrate prevention policies?. Asian J. Psychiatry.

[31] Pirkis J., Gunnell D., Shin S., Del Pozo-Banos M., Arya V., Aguilar P.A., Appleby L., Arafat S.M.Y., Arensman E., Ayuso-Mateos J.L. (2022). Suicide numbers during the first 9–15 months of the COVID-19 pandemic compared with pre-existing trends: An interrupted time series analysis in 33 countries. EClinicalMedicine.

[32] Pirkis J., John A., Shin S., DelPozo-Banos M., Arya V., Analuisa-Aguilar P., Appleby L., Arensman E., Bantjes J., Baran A. (2021). Suicide trends in the early months of the COVID-19 pandemic: An interrupted time-series analysis of preliminary data from 21 countries. Lancet Psychiatry.

[33] Tandon R. (2021). COVID-19 and suicide: Just the facts. Key learnings and guidance for action. Asian J. Psychiatry.

[34] Yan Y., Hou J., Li Q., Yu N.X. (2023). Suicide before and during the COVID-19 Pandemic: A Systematic Review with Meta-Analysis. Int. J. Environ. Res. Public Health.

[35] Okada M. (2022). Is an increase in Japan’s suicides caused by COVID-19 alone?. Asian J. Psychiatry.

[36] Okada M., Matsumoto R., Motomura E., Shiroyama T., Murata M. (2022). Exploring characteristics of increased suicide during the COVID-19 pandemic in Japan using provisional governmental data. Lancet Reg. Health West. Pac..

[37] Matsumoto R., Motomura E., Okada M. (2023). Fluctuation of suicide mortality and temporal causality from unemployment duration to suicide mortality in Japan during 2009–2022. Asian J. Psychiatry.

[38] Matsumoto R., Motomura E., Onitsuka T., Okada M. (2023). Trends in Suicidal Mortality and Motives Among Working-ages Individuals in Japan during 2007–2022. Eur. J. Investig. Health Psychol. Educ..

[39] Sakamoto H., Koda M., Eguchi A., Endo K., Arai T., Harada N., Nishio T., Nomura S. (2024). Excess suicides in Japan: A three-year post-pandemic assessment of gender and age disparities. Psychiatry Res..

[40] Nomura S., Kawashima T., Yoneoka D., Tanoue Y., Eguchi A., Gilmour S., Kawamura Y., Harada N., Hashizume M. (2021). Trends in suicide in Japan by gender during the COVID-19 pandemic, up to September 2020. Psychiatry Res..

[41] Turecki G., Brent D.A., Gunnell D., O’Connor R.C., Oquendo M.A., Pirkis J., Stanley B.H. (2019). Suicide and suicide risk. Nat. Rev. Dis. Primers.

[42] Hughes J.L., Horowitz L.M., Ackerman J.P., Adrian M.C., Campo J.V., Bridge J.A. (2023). Suicide in young people: Screening, risk assessment, and intervention. BMJ.

[43] O’Connor R.C., Kirtley O.J., de Beurs D. (2024). Preventing suicide: Understanding the complex interplay between individual and societal factors. Lancet Public Health.

[44] Chan W.S., Yip P.S., Wong P.W., Chen E.Y. (2007). Suicide and unemployment: What are the missing links?. Arch. Suicide Res..

[45] Chen Y.Y., Yip P.S., Lee C., Fan H.F., Fu K.W. (2010). Economic fluctuations and suicide: A comparison of Taiwan and Hong Kong. Soc. Sci. Med..

[46] Yip K.S., Ng Y.N. (2002). Chinese cultural dynamics of unemployability of male adults with psychiatric disabilities in Hong Kong. Psychiatr. Rehabil. J..

[47] Carrasco-Barrios M.T., Huertas P., Martin P., Martin C., Castillejos M.C., Petkari E., Moreno-Kustner B. (2020). Determinants of Suicidality in the European General Population: A Systematic Review and Meta-Analysis. Int. J. Environ. Res. Public Health.

[48] Milner A., Page A., LaMontagne A.D. (2014). Cause and effect in studies on unemployment, mental health and suicide: A meta-analytic and conceptual review. Psychol. Med..

[49] Milner A., Page A., LaMontagne A.D. (2013). Long-term unemployment and suicide: A systematic review and meta-analysis. PLoS ONE.

[50] Abdou R., Cassells D., Berrill J., Hanly J. (2020). An empirical investigation of the relationship between business performance and suicide in the US. Soc. Sci. Med..

[51] Claveria O., Soric M., Soric P. (2024). Analysis of the impact of financial and labour uncertainty on suicide mortality in England. Health Place.

[52] Goto H., Kawachi I., Vandoros S. (2024). The association between economic uncertainty and suicide in Japan by age, sex, employment status, and population density: An observational study. Lancet Reg. Health West. Pac..

[53] Antonakakis N., Gupta R. (2016). Is Economic Policy Uncertainty Related to Suicide Rates? Evidence from the United States. Soc. Indic. Res..

[54] Vandoros S., Kawachi I. (2021). Economic uncertainty and suicide in the United States. Eur. J. Epidemiol..

[55] Vandoros S., Avendano M., Kawachi I. (2019). The association between economic uncertainty and suicide in the short-run. Soc. Sci. Med..

[56] Claveria O. (2022). Global economic uncertainty and suicide: Worldwide evidence. Soc. Sci. Med..

[57] Okubo R., Matsumoto R., Motomura E., Okada M. (2024). Uncertainties of Economic Policy and Government Management Stability Played Important Roles in Increasing Suicides in Japan from 2009 to 2023. Int. J. Environ. Res. Public Health.

[58] Pirkis J., Dandona R., Silverman M., Khan M., Hawton K. (2024). Preventing suicide: A public health approach to a global problem. Lancet Public Health.

[59] Hawton K., Pirkis J. (2024). Preventing suicide: A call to action. Lancet Public Health.

[60] Okada M., Matsumoto R., Motomura E. (2024). Suicide mortality rates in Japan before and beyond the COVID-19 pandemic era. BMJ Open.

[61] MHLW Basic Data on Suicide in Region. https://www.mhlw.go.jp/stf/seisakunitsuite/bunya/0000140901.html.

[62] Matsumoto R., Kawano Y., Motomura E., Shiroyama T., Okada M. (2022). Analyzing the Changing Relationship Between Personal Consumption and Suicide Mortality During COVID-19 Pandemic in Japan, using governmental and personal consumption transaction databases. Front. Public Health.

[63] MIAC Surveys of Population, Population Change and the Number of Households Based on the Basic Resident Registration. https://www.e-stat.go.jp/en/statistics/00200241.

[64] MHLW Labor Force Survey. https://www.e-stat.go.jp/en/statistics/00200531.

[65] MHLW Survey on the Actual Situation of Disabled Persons. https://www.mhlw.go.jp/toukei/list/111-1.html.

[66] Matsumoto R., Motomura E., Shiroyama T., Okada M. (2023). Impact of the Japanese government’s “general policies for comprehensive measures against suicide” on youth suicide from 2007–2022. BJPsych Open.

[67] Okada M., Matsumoto R., Shiroyama T., Motomura E. (2023). Suicidal Mortality and Motives Among Middle-School, High-School and University Students. JAMA Netw. Open.

[68] RIETI Japan Economic Policy Uncertainty Index. https://www.rieti.go.jp/jp/database/policyuncertainty/index.html.

[69] Baker S.R., Bloom N., Davis S.J. (2016). Measuring Economic Policy Uncertainty*. Q. J. Econ..

[70] Kyo K., Noda H., Kitagawa G. (2022). Co-movement of Cyclical Components Approach to Construct a Coincident Index of Business Cycles. J. Bus. Cycle Res..

[71] Kim H.J., Fay M.P., Feuer E.J., Midthune D.N. (2000). Permutation tests for joinpoint regression with applications to cancer rates. Stat. Med..

[72] Linden A. (2015). Conducting Interrupted Time-series Analysis for Single- and Multiple-group Comparisons. Stata J. Promot. Commun. Stat. Stata.

[73] Bernal J.L., Cummins S., Gasparrini A. (2017). Interrupted time series regression for the evaluation of public health interventions: A tutorial. Int. J. Epidemiol..

[74] Matsumoto R., Motomura E., Okada M. (2024). Impacts of Working Hours, Wages, and Regular Employment Opportunity on Suicide Mortalities of Employed and Unemployed Individuals before and during the COVID-19 Pandemic in Japan. Int. J. Environ. Res. Public Health.

[75] Liu B., Kim H.-J., Feuer E.J., Graubard B.I. (2023). Joinpoint Regression Methods of Aggregate Outcomes for Complex Survey Data. J. Surv. Stat. Methodol..

[76] Li J., Chan N.B., Xue J., Tsoi K.K.F. (2022). Time series models show comparable projection performance with joinpoint regression: A comparison using historical cancer data from World Health Organization. Front. Public Health.

[77] Linden A. (2017). A Comprehensive set of Postestimation Measures to Enrich Interrupted Time-series Analysis. Stata J. Promot. Commun. Stat. Stata.

[78] Yoshioka E., Hanley S.J.B., Sato Y., Saijo Y. (2022). Impact of the COVID-19 pandemic on suicide rates in Japan through December 2021: An interrupted time series analysis. Lancet Reg. Health West. Pac..

[79] Seposo X.T. (2021). COVID-19 threatens decade-long suicide initiatives in Japan. Asian J. Psychiatry.

[80] Ewusie J.E., Blondal E., Soobiah C., Beyene J., Thabane L., Straus S.E., Hamid J.S. (2017). Methods, applications, interpretations and challenges of interrupted time series (ITS) data: Protocol for a scoping review. BMJ Open.

[81] Ewusie J.E., Soobiah C., Blondal E., Beyene J., Thabane L., Hamid J.S. (2020). Methods, Applications and Challenges in the Analysis of Interrupted Time Series Data: A Scoping Review. J. Multidiscip. Healthc..

[82] Hatemi-J A. (2004). Multivariate tests for autocorrelation in the stable and unstable VAR models. Econ. Model..

[83] Yang R., An X., Chen Y., Yang X. (2023). The Knowledge Analysis of Panel Vector Autoregression: A Systematic Review. Sage Open.

[84] Yalta A.T., Cottrell A., Rodrigues P.C. (2024). Computational econometrics with gretl. Comput. Stat..

[85] De Bruin A., Agyemang A., Chowdhury M.I.H. (2019). New insights on suicide: Uncertainty and political conditions. Appl. Econ. Lett..

[86] Matsumoto R., Motomura E., Okada M. (2023). Impacts of Complete Unemployment Rates Disaggregated by Reason and Duration on Suicide Mortality from 2009–2022 in Japan. Healthcare.

[87] OECD Mental Health and Work. https://www.oecd.org/en/publications/mental-health-and-work_5jxsvvn6pq6g-en.html.

[88] Bruggeman H., Héroufosse J., Van der Heyden J., Smith P. (2024). The relationship between mental health and employment: A systematic review of cohort studies. Eur. J. Public Health.

[89] Matsumoto Y., Sakurai H., Aoki Y., Takaesu Y., Okajima I., Tachimori H., Murao M., Maruki T., Tsuboi T., Watanabe K. (2025). Relationship between Severity of Depressive Symptoms and Continuous Employment in Patients with Mood Disorders. Int. J. Neuropsychopharmacol..

[90] Fasching H. (2014). Vocational education and training and transitions into the labour market of persons with intellectual disabilities. Eur. J. Spec. Needs Educ..

[91] Weld-Blundell I., Shields M., Devine A., Dickinson H., Kavanagh A., Marck C. (2021). Vocational Interventions to Improve Employment Participation of People with Psychosocial Disability, Autism and/or Intellectual Disability: A Systematic Review. Int. J. Environ. Res. Public Health.

[92] Jones P.B. (2013). Adult mental health disorders and their age at onset. Br. J. Psychiatry Suppl..

[93] Twenge J.M., Cooper A.B., Joiner T.E., Duffy M.E., Binau S.G. (2019). Age, period, and cohort trends in mood disorder indicators and suicide-related outcomes in a nationally representative dataset, 2005–2017. J. Abnorm. Psychol..

[94] Mithout A.-L. (2021). From equal access to employment to equal career opportunities?. Alter.

[95] Pirkis J., Gunnell D., Hawton K., Hetrick S., Niederkrotenthaler T., Sinyor M., Yip P.S.F., Robinson J. (2023). A Public Health, Whole-of-Government Approach to National Suicide Prevention Strategies. Crisis.

[96] Park S., Hatim Sulaiman A., Srisurapanont M., Chang S.M., Liu C.Y., Bautista D., Ge L., Choon Chua H., Pyo Hong J., Mood Disorders Research A. (2015). The association of suicide risk with negative life events and social support according to gender in Asian patients with major depressive disorder. Psychiatry Res..

[97] Neubaum G., Krämer N.C. (2016). Monitoring the Opinion of the Crowd: Psychological Mechanisms Underlying Public Opinion Perceptions on Social Media. Media Psychol..

[98] Siggiridou E., Koutlis C., Tsimpiris A., Kugiumtzis D. (2019). Evaluation of Granger Causality Measures for Constructing Networks from Multivariate Time Series. Entropy.

