# Supplementary material for: Suicides Mortality of Unemployed Individuals Becomes a Serious Public Health Concern in Japan in Post-COVID-19 Pandemic Era
Source: Int J Environ Res Public Health. 2025 Aug 22;22(9):1315. doi: 10.3390/ijerph22091315 (PMC12469699; doi:10.3390/ijerph22091315)
Supplement: Supplementary file 1 [file ijerph-22-01315-s001.zip › ijerph-3797555-supplementary.pdf]

Supplementary Data List

Supplementary Table S1: APC (±95%CI) of CMR-suicides of total, employed and unemployed males and females of overall-ages from 2009-2024 using JPRA (p2)
Supplementary Table S2: APC (±95%CI) of CMR-suicides of total working-age males and females from 2009-2024 using JPRA. (p2)
Supplementary Table S3: APC (±95%CI) of CMR-suicides of employed working-age males and females from 2009-2024 using JPRA. (p3)
Supplementary Table S4: APC (±95%CI) of CMR-suicides of unemployed working-age males and females from 2009-2024 using JPRA. (p3)
Supplementary Figure S1: Fluctuations of CMR-suicides of total and employed males and females of overall ages from 2009-2024 using ITSA.(p4)
Supplementary Figure S2: Fluctuations of CMR-suicides of 40-69 total males from 2009-2024 using ITSA.(p4)
Supplementary Figure S3: Fluctuations of CMR-suicides of 40-69 employed males from 2009-2024 using ITSA.(p5)
Supplementary Figure S4: Fluctuations of CMR-suicides of 30-59 employed females from 2009-2024 using ITSA.(p5)

Table S1: APC (±95%CI) of CMR-suicides of total, employed and unemployed males and females of overall-ages from 2009-2024 using JPRA

Total males				
year	APC	lowerCI	upperCI	
2009-2016	-0.604	-0.667	-0.541	**
2016-2021	0.033	-0.087	0.153	
2021-2023	0.737	-0.388	1.874	
2023-2024	-0.924	-1.514	-0.331	**

Total females				
year	APC	lowerCI	upperCI	
2009-2019	-0.432*	-0.474	-0.391	**
2019-2020	3.042*	0.564	5.580	*
2020-2024	-0.338*	-0.514	-0.161	**

Employed males				
year	APC	lowerCI	upperCI	
2009-2016	-0.700	-0.776	-0.625	**
2016-2022	0.170	0.067	0.274	**
2022-2023	3.421	-10.717	19.798	
2023-2024	-1.391	-2.069	-0.709	**

Employed females				
year	APC	lowerCI	upperCI	
2009-2016	-0.593	-0.685	-0.502	**
2016-2019	0.260	-0.134	0.655	
2019-2020	3.343	0.011	6.786	**
2020-2024	-0.271	-0.505	-0.036	*

Employed males				
year	APC	lowerCI	upperCI	
2009-2017	-0.890*	-1.012	-0.768	**
2017-2018	4.413	-0.694	9.783	
2018-2021	-1.077*	-1.647	-0.503	**
2021-2022	8.427*	3.124	14.003	**
2022-2024	-0.577	-1.357	0.209	

Employed females			
year	APC	lowerCI	upperCI
2009-2021	-0.130	-0.275	0.016
2021-2022	13.671*	1.878	26.829
2022-2024	-0.954	-2.562	0.681

Table S2: APC (±95%CI) of CMR-suicides of total working-age males and females from 2009-2024 using JPRA.

Males (30-39)				
year	APC	lowerCI	upperCI	
2009-2016	-0.536*	-0.629	-0.442	**
2016-2024	0.139*	0.043	0.235	**

Females (30-39)				
year	APC	lowerCI	upperCI	
2009-2019	-0.546*	-0.615	-0.477	**
2019-2020	4.282*	0.141	8.594	*
2020-2024	-0.373*	-0.667	-0.079	*

Males (40-49)				
year	APC	lowerCI	upperCI	
2009-2016	-0.765*	-0.841	-0.689	**
2016-2022	0.151*	0.029	0.274	*
2022-2023	3.736	-11.485	21.574	
2023-2024	-1.070*	-1.862	-0.272	**

Females (40-49)				
year	APC	lowerCI	upperCI	
2009-2019	-0.476*	-0.535	-0.416	**
2019-2020	3.864*	0.297	7.558	*
2020-2024	-0.624*	-0.877	-0.372	**

Males (50-59)				
year	APC	lowerCI	upperCI	
2009-2013	-0.976*	-1.141	-0.811	**
2013-2021	-0.274*	-0.358	-0.190	**
2021-2022	3.629	-1.956	9.532	
2022-2024	-0.863*	-1.298	-0.427	**

Females (50-59)				
year	APC	lowerCI	upperCI	
2009-2019	-0.321*	-0.377	-0.265	**
2019-2020	2.807	-0.596	6.326	
2020-2024	-0.339*	-0.598	-0.080	*

Males (60-69)				
year	APC	lowerCI	upperCI	
2009-2016	-0.827	-0.915	-0.738	**
2016-2021	-0.138	-0.300	0.024	
2021-2023	1.218	-0.344	2.805	
2024-2024	-0.944	-1.760	-0.120	*

Females (60-69)				
year	APC	lowerCI	upperCI	
2009-2019	-0.590*	-0.644	-0.536	**
2019-2020	2.882	-0.334	6.203	
2020-2024	-0.401*	-0.631	-0.170	*

Table S3: APC (±95%CI) of CMR-suicides of employed working-age males and females from 2009-2024 using JPRA.

Males (30-39)				
year	APC	lowerCI	upperCI	
2009-2015	-0.476	-0.602	-0.442	**
2015-2024	0.186	0.101	0.235	**

Females (30-39)				
year	APC	lowerCI	upperCI	
2009-2016	-0.532	-0.691	-0.372	**
2016-2019	-0.201	-0.909	0.513	
2019-2020	4.734	-1.053	10.861	
2020-2024	-0.201	-0.601	0.200	

Males (40-49)				
year	APC	lowerCI	upperCI	
2009-2016	-0.778	-0.868	-0.687	**
2016-2021	0.338	0.208	0.468	**
2021-2023	3.560	-13.402	23.844	
2023-2024	-1.297	-2.187	-0.399	**

Females (40-49)				
year	APC	lowerCI	upperCI	
2009-2016	-0.515	-0.680	-0.349	**
2016-2019	0.395	-0.160	0.953	
2019-2020	8.173	-22.478	50.942	
2020-2024	-0.465	-0.886	-0.043	*

Males (50-59)				
year	APC	lowerCI	upperCI	
2009-2015	-0.867	-0.990	-0.744	**
2015-2021	-0.117	-0.263	0.028	
2021-2022	2.630	-0.862	6.244	
2022-2024	-1.093	-1.688	-0.494	**

Females (50-59)				
year	APC	lowerCI	upperCI	
2009-2016	-0.446	-0.609	-0.283	**
2016-2019	0.117	-0.427	0.663	
2019-2020	7.994	-22.217	49.940	
2020-2024	-0.225	-0.627	0.179	

Males (60-69)				
year	APC	lowerCI	upperCI	
2009-2015	-1.094	-1.222	-0.967	**
2015-2021	-0.085	-0.246	0.076	
2021-2023	1.462	-0.248	3.202	
2023-2024	-1.197	-2.186	-0.199	*

Females (60-69)				
year	APC	lowerCI	upperCI	
2009-2017	-0.943	-1.168	-0.717	**
2017-2024	0.214	-0.010	0.439	

Table S4: APC (±95%CI) of CMR-suicides of unemployed working-age males and females from 2009-2024 using JPRA.

Males (30-39)				
year	APC	lowerCI	upperCI	
2009-2017	-0.960*	-1.214	-0.705	**
2017-2024	0.797*	0.459	1.137	**

Females (30-39)				
year	APC	lowerCI	upperCI	
2009-2018	-1.340*	-2.652	-0.010	**
2018-2019	-22.116	-76.111	153.921	
2019-2024	7.321*	4.471	10.249	**

Males (40-49)				
year	APC	lowerCI	upperCI	
2009-2017	-1.091*	-1.289	-0.891	**
2017-2018	2.041	-0.355	4.496	
2018-2021	-1.328*	-2.337	-0.310	*
2021-2022	9.548*	1.637	18.075	*
2022-2024	-1.356*	-2.51	-0.188	*

Females (40-49)				
year	APC	lowerCI	upperCI	
2009-2021	-1.070*	-1.945	-0.186	*
2021-2023	6.544*	1.392	11.957	*
2023-2024	-3.013	-15.279	11.029	

Males (50-59)				
year	APC	lowerCI	upperCI	
2009-2017	-0.983*	-1.176	-0.789	**
2017-2019	1.946*	0.310	3.608	*
2019-2021	-1.643*	-2.893	-0.376	*
2021-2022	7.471	0.075	15.585	*
2022-2024	-0.611	-1.741	0.531	

Females (50-59)				
year	APC	lowerCI	upperCI	
2009-2021	-1.439*	-2.525	-0.341	*
2021-2022	16.004	2.699	38.301	*
2022-2024	-1.134	-10.864	9.658	

Males (60-69)				
year	APC	lowerCI	upperCI	
2009-2018	-1.287*	-1.560	-1.013	**
2018-2019	9.395	-3.589	24.127	
2019-2021	-3.272*	-5.287	-1.213	**
2021-2022	18.754*	4.66	34.747	**
2022-2024	-0.828	-2.776	1.159	

Females (60-69)				
year	APC	lowerCI	upperCI	
2009-2021	-4.106	-2.047	10.647	
2021-2022	64.884	0.156	289.246	*
2022-2024	-3.914	-15.489	-0.247	*

Figure S1: Fluctuations of CMR-suicides of total and employed males and females of overall ages from 2009-2024 using ITSA.

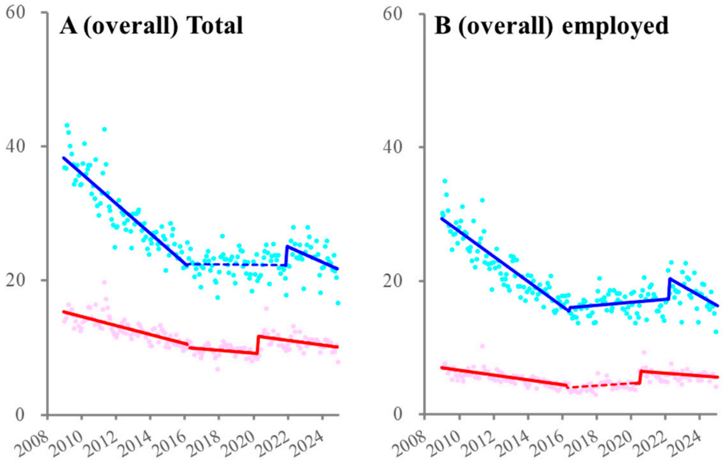

Fluctuation of CMR-suicides of total males/females (A) and employed males/females (B) from January/2009 to December/2024 in Japan using ITSA. Ordinate and abscissa indicate the annualized monthly CMR-suicides (per 100,000 population) and calendar years, respectively. Blue and red indicate CMR-suicides of males and females, respectively. Circles indicate the observed monthly CMR-suicides. Solid and dotted lines indicate the significant and non-significant trends of CMR-suicides, respectively. Intervention periods for ITSA were set at April/2016, April/2020 and December/2021.

Figure S2: Fluctuations of CMR-suicides of 40-69 total males from 2009-2024 using ITSA.

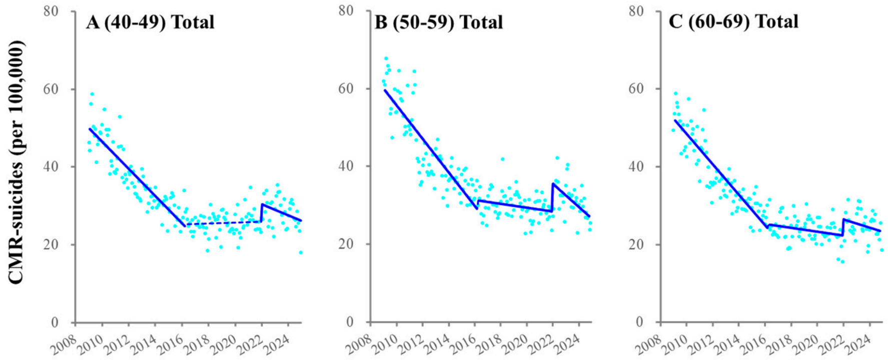

Fluctuation of CMR-suicides of total males 40-49 years of age (A), 50-59 (B) and 60-69 (C) from January/2009 to December/2024 in Japan using ITSA. Ordinate and abscissa indicate the annualized monthly CMR-suicides (per 100,000 population) and calendar years, respectively. Circles indicate the observed monthly CMR-suicides. Solid and dotted lines indicate the significant and non-significant trends of CMR-suicides, respectively. Intervention periods for ITSA were set at April/2016 and December/2021.

Figure S3: Fluctuations of CMR-suicides of 40-69 employed males from 2009-2024 using ITSA.

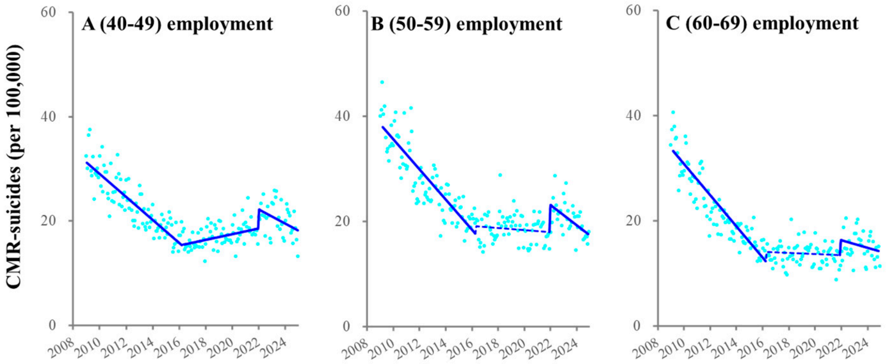

Fluctuation of CMR-suicides of employed males 40-49 years of age (A), 50-59 (B) and 60-69 (C) from January/2009 to December/2024 in Japan using ITSA. Ordinate and abscissa indicate the annualized monthly CMR-suicides (per 100,000 population) and calendar years, respectively. Circles indicate the observed monthly CMR-suicides. Solid and dotted lines indicate the significant and non-significant trends of CMR-suicides, respectively. Intervention periods for ITSA were set at April/2016 and December/2021.

Figure S4: Fluctuations of CMR-suicides of 30-59 employed females from 2009-2024 using ITSA.

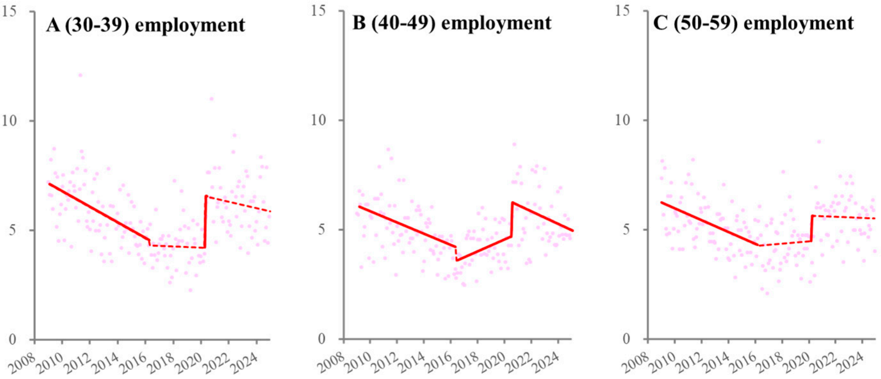

Fluctuation of CMR-suicides of employed females 30-39 years of age (A), 40-49 (B) and 50-59 (C) from January/2009 to December/2024 in Japan using ITSA. Ordinate and abscissa indicate the annualized monthly CMR-suicides (per 100,000 population) and calendar years, respectively. Circles indicate the observed monthly CMR-suicides. Solid and dotted lines indicate the significant and non-significant trends of CMR-suicides, respectively. Intervention periods for ITSA were set at April/2016 and April/2020.
